# Assessment of Risk Factors of African Swine Fever in India: Perspectives on Future Outbreaks and Control Strategies

**DOI:** 10.3390/pathogens9121044

**Published:** 2020-12-12

**Authors:** Mousumi Bora, Durlav Prasad Bora, Mohan Manu, Nagendra Nath Barman, Lakshya Jyoti Dutta, Pesingi Pavan Kumar, Suvaneeth Poovathikkal, Kuralayanapalya Puttahonnappa Suresh, Ramadevi Nimmanapalli

**Affiliations:** 1 Faculty of Veterinary and Animal Sciences, Banaras Hindu University, Mirzapur 231001, India; mousumeebora11@gmail.com (M.B.); mmanu@bhu.ac.in (M.M.); pesingi@bhu.ac.in (P.P.K.); suvaneeth@bhu.ac.in (S.P.); ramadevin@bhu.ac.in (R.N.); 2 College of Veterinary Science, Assam Agricultural University (AAU), Guwahati 781022, India; nagendra.barman@aau.ac.in (N.N.B.); lakhyajyoti.dutta@aau.ac.in (L.J.D.); 3 National Institute of Veterinary Epidemiology and Disease Informatics (NIVEDI), Bengaluru 560064, India; Suresh.KP@icar.gov.in

**Keywords:** African swine fever virus, outbreak, domestic pigs, wild-life reservoirs, risk assessment, control strategies

## Abstract

African swine fever (ASF) is one of the most important transboundary diseases of pigs. ASF has been identified in India for the first time in domestic pigs from outbreaks reported in two of the northeastern states, Arunachal Pradesh and Assam in 2020. A total of 11 ASF outbreaks in different regions killed over 3700 pigs and devastated the economy of small-scale livestock owners of both the states. Considering the first outbreak of ASF in India, a generic risk assessment framework was determined to identify potential risk factors that might favor future emergence of the disease. Based on the Indian scenario, we considered population density of host, farming practice, availability of biological vectors and wildlife reservoirs, epidemiological cycles, and international trade to analyze the possibility of future outbreaks of ASF and chances of establishment of endemism. On critical analysis of the identified risk factors associated with ASFV transmission, we observed that the risk factors are well preserved in the Indian geography and might participate in future outbreaks, further disseminating the disease to nearby countries. Since no vaccine is currently available against ASF, the domestic and the wild pigs (wild boars and the endangered pygmy hogs native to India) of this region are under constant threat of infection. For the near future, this region will have to continue to rely on the implementation of preventive measures to avoid the devastating losses that outbreaks can cause. The various adaptive control strategies to minimize the risks associated with the transmission of ASF, keeping our views to Indian settings, have been described. The risk-analysis framework presented in the study will give a further understanding of the dynamics of disease transmission and will help to design control strategies and corresponding measures to minimize the catastrophic consequences of ASF disease.

## 1. Introduction

African swine fever (ASF) is a highly fatal viral infectious disease that manifests as a hemorrhagic fever in affected pigs [1]. ASF has been listed as a priority disease in the World Organization for Animal Health (OIE), owing to its socio-economic importance and transboundary potential [2]. The first report of ASF was detected in Kenya as a disease entity distinct from classical swine fever (CSF) [3]. Since its first inception, ASF has spread from its historic range of Africa, to several countries in Europe (France, Italy, Malta, Belgium, and the Netherlands and Iberian Peninsula) [1,4]. Although Europe has successfully eradicated ASF in 1995 with an exception in Sardinia, the second phase of ASF in East Europe in 2007 and its recent spread to Asia created devastating effects, threatening both domestic and wildlife conservation [5]. The disease ASF is caused by African swine fever virus (ASFV) of genus *Asfivirus* and is closely related to a novel asfarvirus (Abalone asfa-like virus) identified within the family *Asfarviridae* [6]. ASFV infects domestic and wildswine (*Sus scrofa*), warthogs (*Phacochoerus africanus; Phacochoerus aethiopicus*), and bush pigs (*Potamochoerus porcus*) [1]. ASFV can be transmitted directly during (i) contact between infected and susceptible pigs; (ii) consumption of the meat from infected pigs; (iii) bites of infected soft ticks (Ornithodoros species); and indirectly (iv) by contact with fomites contaminated by virus-containing matters such as blood, feces, urine, or saliva from infected pigs [1]. Clinical disease of ASF can manifest in multiple ways, ranging from peracute (mortality ~100%) to an asymptomatic infection [4]. Acute infections are typically characterized by a high fever, anorexia, lethargy, weakness, recumbency, diarrhea and/or constipation, abdominal pain, hemorrhagic signs, respiratory distress, nasal and conjunctival discharge, and abortions in pregnant females, followed by death within 6–13 days after the onset of clinical signs [7]. Subacute infections are often recorded with high mortality in young animals and include clinical signs such as abortion, fever, and transient hemorrhage with death or recovery within 3–4 weeks [1,4]. Chronic infections are associated with low mortality and clinical signs such as intermittent or low fever, swelling of joints, loss of appetite, and depression which might develop over 2–15 months [7,8].

India has reported the first outbreak of ASF in the domestic pig population of two of the northeastern (NE) states viz. Arunachal Pradesh and Assam to OIE on 21 May 2020 [9]. The NE region of India, owing to its unique geographical location while sharing international boundaries with China, Bhutan, Bangladesh, and Myanmar, bears a constant threat of transboundary emerging diseases though its porous borders [10]. Apart from the first outbreak of ASF in NE India, this region has also reported several other emerging diseases of pigs such as porcine reproductive and respiratory syndrome [11] and porcine circovirus-2 infections [12]. In this article, the authors will discuss the risk factors/indicators that might favor the spread of ASF infections in India, its possibilities of establishment of endemic infections, and the control strategies to prevent future outbreaks.

## 2. First Emergence of ASF in India

An unusual mortality in pigs was reported earlier on January 2020 from several districts of Assam (Districts: Dhemaji, Biswanath, Sivasagar, Jorhat, and Dibrugarh) as well as from Arunachal Pradesh (Districts: Papumpare and East Siang). The striking feature of the outbreaks in both the states was high mortality (100%) in affected group of pigs irrespective of the age groups. After scrutiny of the clinical case reports, samples were initially screened for CSF, owing to its endemic status in India and later for ASF as well, considering the recent emergence of ASF in the neighboring countries like China and other Asian countries. The tests were performed at the College of Veterinary Sciences and North Eastern Regional Disease Diagnostic Laboratory (NERDDL), Guwahati with support from ICAR-National Research Centre on Pigs, Assam and ICAR Research Complex for NEH Region, Meghalaya. As the tissue samples of pigs collected from the outbreaks in Assam indicated the presence of ASFV in PCR, samples from both Assam and Arunachal Pradesh were sent to the OIE reference laboratory, ICAR-National Institute for High Security Animal Diseases (NIHSAD), Bhopal for further confirmation. ICAR-NIHSAD has confirmed the presence of ASFV in the samples through successful isolation of the virus in cell culture, further confirming the reported ASFV variant to be of genotype II origin [9].

## 3. Etiology and Natural Reservoirs of ASF

ASF is caused by ASFV, a DNA arbovirus belonging to the family *Asfarviridae* [4]. ASFV is an enveloped virus with icosahedral morphology and an average diameter of 200 nm [13]. The genome consists of a linear double-stranded DNA molecule of 170–193 kbp depending on the isolate with terminal inverted repeats and hairpin loops [14]. To date, 24 distinct genotypes of ASFV have been described based on sequencing of C-terminal B646L gene that encodes the p72 protein (major structural protein) [15]. The virus primarily infects cells of the mononuclear phagocytic system (monocytes and macrophages) and replicates in the cytoplasm [16]. All age groups from the members of the family *Suidae* are susceptible to ASFV infection [17,18].

ASF can lead to high mortalities in domestic pigs while being asymptomatic in the natural suid reservoir hosts [19]. Wild pigs (warthogs and bush pigs) and soft ticks of the genus Ornithodoros serve as natural reservoirs in ASFV transmission [20]. Wild pigs including warthogs and bush pigs may be persistently infected, generally developing asymptomatic infections, often referred to as the sylvatic cycle [21]. Young warthogs may develop a transient viremia to infect new ticks without developing clinical disease, and adult warthogs may be resistant to the pathogenic effects of the virus, although the virus can be often extracted from their lymph nodes [22,23]. Infection from the wild pigs to the domestic population is largely dependent on ticks of the Ornithodoros species than infections through contact between wild and domestic pigs [24,25]. Apart from soft ticks, biting flies (*Stomoxys* spp.) have shown to be capable of transmitting the disease into the swine population [26].

## 4. Epidemiological Cycles of ASF

The epidemiology of ASF was described as comprising four independent epidemiologic cycles: (i) sylvatic, (ii) tick–pig, (iii) domestic, and (iv) wild boar–habitat cycle (a newly discovered cycle in Europe) [27]. The sylvatic cycle, mostly restricted to the African continent, is maintained between Ornithodoros ticks and natural reservoirs (warthogs and bush pigs) that are resistant to the ASFV and usually do not develop any clinical disease [28,29]. In the tick–pig cycle, the virus is transmitted among domestic pigs through ticks serving as a reservoir and allowing the virus to persist locally in the environment [30]. In the domestic cycle, the virus is transmitted among domestic pigs or from pig products to domestic pigs without the involvement of natural reservoirs [31]. The wild boar–habitat cycle is a newly described cycle in Central and Eastern Europe, while investigating the epidemiological pattern of ASF infections in wild and domestic pig population [27]. This newly evolved epidemiological cycle involves wild boar (*Sus scrofa*), the wild boar habitat, and their carcasses for the maintenance and transmission of ASFV to domestic pigs [27]. The wild boar–habitat cycle may describe the possibilities of new infections in areas with wild boar population and in the interspaces between wild and domesticated swine habitat such as a wildlife sanctuary or a national park.

## 5. Risk Identification and Assessment of ASF

Considering the possibility of ASFV to cause future outbreaks and establishment of endemism in India, several factors that exert selection pressures on emergence of new animal diseases need to critically analyzed. We identified some of the risk factors/indicators in Indian context which might serve as a connection for further emergence of ASF in this region. The identified risk factors of ASF are described critically, keeping in view the social and ecological understanding of agriculture and food security of the human populations which depends on agriculture and livestock for their living.

### 5.1. Population Density of Domestic Pigs

Among the livestock species, pigs find an important place in the Indian economy as it not only contributes to the livelihood security of rural masses but also improves the socio-economic status of the marginal farmers and the weaker section of the society [32]. The northeastern (NE) region of India mostly comprises of a high proportion of tribal people where backyard pig farming is an integral part of their way of living [33]. Apart from NE India, pig farming has been observed in several states of the mainland such as Jharkhand, West Bengal, and Uttar Pradesh, especially among the down trodden and tribal population as a backyard subsidiary enterprise [34,35,36,37]. According to the latest livestock census report (2019), the current pig population is 9.06 million (M), the highest being in the state of Assam (2.10 M) followed by Jharkhand (1.28 M), Meghalaya (0.71 M), West Bengal (0.54 M), and Chhattisgarh (0.53 M) [38]. Over 70% of the pigs reared in India are of indigenous origin [39]. According to studies related to infectious diseases and group sizes, it has been found that outbreaks of any disease reach higher prevalence when groups are larger and dense [40]. The majority of the marginal families involved in backyard farming usually keep an average of 2–3 indigenous or crossbred pigs for fattening with zero to minimum inputs in terms of family labor and feeding [41,42,43]. Pig owners, especially from remote and rural settings, are observed to show keen interest in small-scale pig farming (10–15 pigs per family), mostly with an aim to get avenues for additional income and savings for their children’s education and medical treatment based on the locally available resources [36]. Even though pigs maintained in such locations are clustered into smaller groups, the rural households are connected close to each other, thus predisposing the animals to infectious animal diseases and their spread to closely reared/in-contact animals.

**Figure 1:**
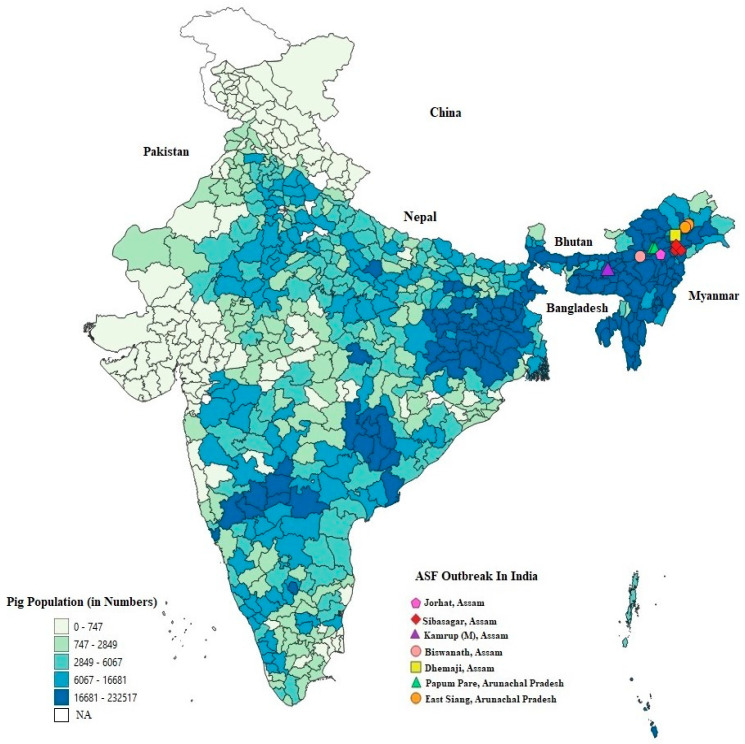
Geospatial map representing district-wise domestic pig population of India composed and exported using QGIS Software. The data on population statistics of pigs has been adapted from 20th Livestock census report of India, 2019. The featured outbreaks of African swine fever in the Indian states of Arunachal Pradesh and Assam have been adapted from the World Organization for Animal Health (OIE), 2020.

To gain a better understanding of the Indian pig scenario, we have analyzed the geographical distribution of district wise domestic pig population of India using statistical data adapted from the 20th livestock census report, 2019 [38]. It was observed that the population of pigs is highest in the NE region followed by the eastern, southern, central, certain parts of northern and western India (Figure 1). Meanwhile, tracking down the first outbreaks of ASF in the NE region of India, it was observed that the outbreaks were reported alongside the river tributaries of Brahmaputra, a transboundary river which flows through Tibet, India, and Bangladesh. The Brahmaputra receives a number of tributaries that flows through several national parks and wildlife sanctuaries of NE region of India, comprising of a significant number of the wild boar population. Therefore, incursion of ASF into the densely distributed domestic pig population of this region, mostly reared under backyard and scavenging systems with poor biosecurity measures, is a major threat to the wild boars and can swipe out the pig population of the entire region if not appropriately controlled. From the NE pocket, the disease may further spread to eastern India and then to the mainland through the river routes and interstate movement of pigs for trade and commerce. Therefore, considering the existing high density of domestic pig population in India, there is every chance that ASF might attain an endemic status if not controlled critically.

### 5.2. Farming Practice

Pig farming in India is primarily an unorganized small-scale and backyard enterprise characterized by subsistence, low input-low output, and technologically lagged activity dominated by small land holders [33,36]. In the majority of pig rearing areas in India, farmers follow a production system mostly under scavenging conditions, which depends on locally available unconventional feed like plants and limited amount of kitchen waste [44,45]. Scavenging pigs can be found on both urban and rural areas of India where pigs are permitted to scavenge for feed during the day time around the household, streets, and nearby forests, and then allowed to take rest in small enclosures at night. The present outbreaks of ASF reported from NE region of India were detected mostly from remote villages where the scavenging system of rearing of pig is predominant and animals are allowed to move freely around the home in search of feed (free-grazing). Besides, swill feeding (garbage/food scraps) is a common practice throughout India, often concentrated around metropolitan centers as it is economical for pig rearing and production [46,47]. However, in most village settings, swills or feed wastes are not heat treated before feeding pigs and thus remains as a source of infection to the healthy pig population. A study conducted by a group of researchers in the pig production system in mountainous regions of NE India reported pigs that died of diseases are disposed either by burial or dumbed in nearby jungles [44]. The farmers in rural settings lack awareness on infectious diseases and their mode of transmissions. In general, veterinarians are not informed by livestock owners and most of the infectious diseases of animals goes unreported.

**Figure 2:**
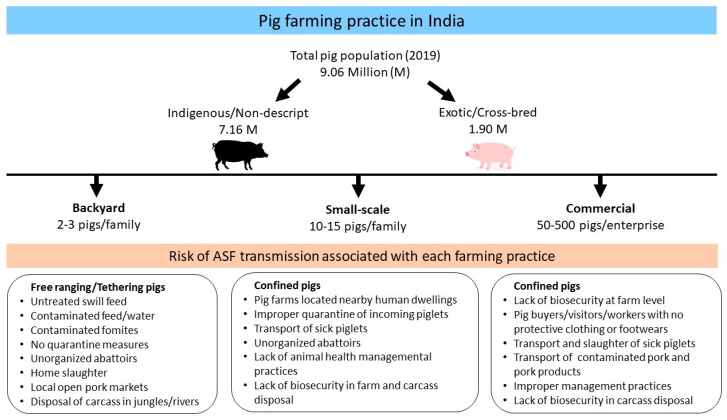
Risk of African swine fever transmission associated with different pig farming practice in India.

Small-scale confined pig production is usually done by marginal farmers and groups of household women for both subsistence and commercial reasons. Here, pigs are confined to pens made of local materials to more modern housing systems and fed with leaves, crop residues, agricultural by-products, or prepared feed. The farmers owning such small-scale pig farms often search for traders within their marketing chain. The traders usually travel between villages and collect pigs to bring them to live animal markets or slaughter areas where mixing of animals at wet markets and during transport is more frequent [45]. Therefore, diseases confined to a group of pigs are more likely to spread to different regions due to the purchase of infected piglets/pigs from unknown sources. Besides, it is not a common practice to quarantine the newly introduced animals in the farms. Moreover, in most rural and remote areas, there are no organized slaughterhouses or abattoirs and pigs are usually slaughtered in home premises or in open meat markets. Thus, sewage from these poorly equipped facilities is directly accessible to other animals as a source of food. Therefore, it is obvious that highly infectious diseases like ASF can easily spread at the local level associated with free-range pig production, local pig movements, and lack of basic biosecurity measures among the pig owners. Commercial pig farms involved in high scale pig production in India are limited and can be found in urban and peri-urban areas where agricultural and market opportunities are available [48]. The risks of transmission of ASFV associated with the pig farming practice in Indian settings are described in Figure 2.

### 5.3. Availability of Tick Vectors

Previous studies have shown that the infected Ornithodoros ticks are able to retain ASFV for long periods, thus serving as a biological vector, and transmit it to susceptible hosts [49,50]. In addition, members of the Ornithodoros species can transmit ASFV from tick to tick through transstadial [51], sexual, and transovarial transmission [52], allowing the virus to persist even in the absence of viremic hosts. Endemism of ASF in any country depends upon the prevalence and geographical distribution of Ornithodoros species [53]. In India, the prevalence of soft ticks has been found to be comparatively lower than hard ticks [54]. This might be because of limited studies on soft ticks, especially on Ornithodoros species, to even identify their geographical distribution. Only a few case reports on tick borne human relapsing fever have been reported from Indian states of Kashmir [55], Madhya Pradesh [56], and Karnataka [57], confirming the involvement of Ornithodoros ticks in their transmission. The present outbreak of ASF in India is still not known in the context of transmission involving tick vectors/biting flies/infective secretions and tissues or possibility of indirect transmission by fomites. Since Ornithodoros species have already been found to be associated with other diseases and its prevalence has been reported from Gujarat [52,58], Nilgiri Hills and the adjoining areas of Tamil Nadu [59], there may be a possibility of involvement of Ornithodoros species with the present outbreak of ASF in NE India. The paucity of research on prevalence of Ornithodoros ticks and their probable role in the transmission of various diseases of animal and public health importance in the country might further enhance the possibility of future outbreaks of ASF in other pig rearing states of India.

### 5.4. Availability of Wildlife Reservoirs and Their Habitat

AFSV is maintained in a stable equilibrium with its wildlife hosts, warthogs, and soft ticks of Ornithodoros species, in a unique ecological niche [60]. Though the sylvatic cycle of ASFV in warthogs contrasts with the domestic cycle in swine, in both instances, soft ticks of the genus Ornithodoros in warthog burrows and domestic pigpens serve as biological hosts and vectors [61]. In wildlife reservoirs, warthogs are native to Africa and often found in abundance [62]. The common warthog (*Phacochoerus africanus*) is widely distributed over sub-Saharan Africa, and has expanded its geographic range to west Africa eastwards to Eritrea and Ethiopia, southward through eastern Africa, and over much of southern Africa to southern Angola, Botswana, and Mozambique to northeast South Africa [62]. The desert warthog (*Phacochoerus aethiopicus*) is presently known only from southeastern Ethiopia, western Somalia, and in central and eastern Kenya [63]. Since warthogs are confined mostly to Africa, outbreaks of ASF recorded outside the continent (recent outbreaks in Europe and Asia including India) raises questions about the involvement of sylvatic cycles, but might have arised through other means of disease dissemination such as infected pig-derived products and circulation of Ornithodoros species across the borders. However, distribution of warthogs (desert warthogs in particular) is largely unstudied for which further field surveys are needed to better determine geographic limits, area of occupancy, abundance, and the impacts of various human/livestock-raising activities on distribution and abundance [63]. There are also limited investigations on bush pigs to understand their contribution in the epidemiology of ASF and the interactions between these natural hosts and domestic pigs [64].

### 5.5. Correlation between the Epidemiological Cycles of ASF

Possibility of future outbreaks or establishment of endemism of ASF in India will depend upon the maintenance of epidemiological cycles of ASF as well as distribution of reservoirs and susceptible hosts. We have analyzed the four established epidemiological cycles of ASF from an Indian perspective to describe the risk associated with each cycle.

#### 5.5.1. Sylvatic Cycle

Sylvatic cycle is maintained between the natural reservoirs and biological vectors of ASFV without causing disease in the reservoir hosts [65]. This cycle is not likely to occur in the Indian scenario because of absence of spatial distribution of the natural reservoirs i.e., warthogs and bush pigs. Even though studies have shown the prevalence of Ornithodoros ticks in India, absence of reservoir hosts from the epidemiological triad will avert perpetuation of ASFV in the environment, thus suppressing the importance of the sylvatic cycle.

#### 5.5.2. Tick–Pig Cycle

The maintenance of the tick–pig cycle can be related to the sylvatic cycle in regions where natural reservoirs of the virus are present and ticks serve as a transmitting medium to the domestic population from the wild reservoir habitat [31,66]. This cycle can still be maintained by transboundary migration of ticks harboring the virus and allowing it to persist locally in the environment. The distribution of soft ticks across the world is not limited to a certain bio-geographic region and has been found in new ecological environments by the dissemination of immature phases by migratory birds and other climatic factors [50]. Dissemination of soft ticks from neighboring countries with similar climatic, socio-economic, and demographic characteristics will always pose a threat for future emergence of ASF in India. Therefore, investigations related to soft tick distribution modeling in the country is extremely crucial to prevent disease outbreaks.

#### 5.5.3. Domestic Cycle

In this cycle ASFV is transmitted from contaminated pig products to domestic pigs by the involvement of human activities such as trade and slaughter [31]. The outbreak of ASF in India can be related to the domestic cycle, where domestic pigs might have attained the infection by means of potential environmental contamination with ASFV through virus escape from infected populations of a nearby country. Maintenance of the domestic cycle endemism in the infected zones and incursion into a different location within the Indian geographic setting is possible because, in most rural settings, pig owners are not aware of the basic biosecurity measures in the farm level. Pigs, while in the incubation period of an infectious disease, are often taken to live animal markets unintentionally or being unaware of disease symptoms. Moreover, the slaughter house waste management system is very poor in India [39]. Unavailability of organized slaughter houses in rural and peri-urban premises makes effective management of waste difficult. The contaminated waste generated from such slaughter houses or pig markets will remain as a source of infection to susceptible animals in nearby regions.

#### 5.5.4. Wild Boar–Habitat Cycle

Wild boar is one of the most widespread group of mammals, which can be found on every continent except Antarctica [67]. The Indian crested wild boar (*Sus scrofa cristatus*) is seen in most of the wildlife protection sanctuaries and is widely distributed in India, Sri Lanka, Nepal, Thailand, and Myanmar [68]. Wild boars exhibit a home range behavior, in which the movements of these animals are generally restricted to a defined area over an extended period of time. However, depending upon ecological conditions, these animals may roam about widely in search of better forage conditions [69,70]. Presently, the wild boar population in India is fragmented and can be observed in isolated groups. Some of these isolated populations can become locally overabundant and depend upon agricultural crops, especially in and around the protected areas and village interface areas for a major part of their food requirement [71]. While utilizing the agro-ecosystem for food, resource, and shelter, contaminated food waste originating from the infected domestic pigs can initiate an ASF epidemic which may accidentally be released into a wild boar habitat. Infections disseminated to a group of wild boar will persist in the wild boar habitat and their carcasses. This cycle will continue to infect healthy wild boar and domestic habitat. Besides, as discussed earlier, in most of the Indian pig rearing states, carcasses or contaminated materials of infected domestic pigs are usually disposed in jungles or thrown in rivers. By doing so, it can not only be a source of infection to natural host and wildlife reservoirs, but also to pygmy hog population (*Porcula salvania*), an endangered species native to India and the only known population that lives in Assam which is estimated to be less than 250 in numbers [72]. The susceptibility of pygmy hogs to ASFV is unclear, although Classical swine fever virus (CSFV), an RNA virus that causes a disease with similar clinical signs to ASFV, can infect and kill pygmy hogs [73].

Based on the epidemiological cycles, the tick–pig, the domestic, and the wild boar–habitat cycles are important in emergence of future outbreak of ASF and should be critically investigated to identify the transmission patterns and to implement control strategies.

### 5.6. International Trade

At the global level, international trade, despite bringing potential health benefits through economic growth, is one of the major driving factors of emerging diseases of animals as well as humans [74]. At present, ASF is a global threat to food security and economic stability of nations [75]. Already, China, a home to half the world’s pig population has lost a third of its pigs (about 100 M) to the devastating outbreak of ASF in the year 2018 and estimated to have led to a direct economic loss of one trillion yuan (approximately 142 billion USD) [76]. Besides China, the country Vietnam, where the pork industry is worth approximately 4 billion USD, has confirmed several outbreaks of ASF in recent years [75]. In India, pork production is limited, representing only 9% of the country’s animal protein sources. Production of pork is concentrated only in few India states and mainly in the NE region of the country, primarily consisting of backyard and informal sector producers [39]. As per Government of India reports, the total pork production in the year 2014–2015 was 464.11 thousand metric tons. The Indian market for processed pork products is small, and the majority of this market is supplied through imports by Belgium, Sri Lanka, Spain, Italy, and the Netherlands in recent years [39,77]. India’s global interaction in import of pork and pork products and human movements in dealing pigs might spread infectious diseases like ASF to emerge further outbreaks. 

**Figure 3:**
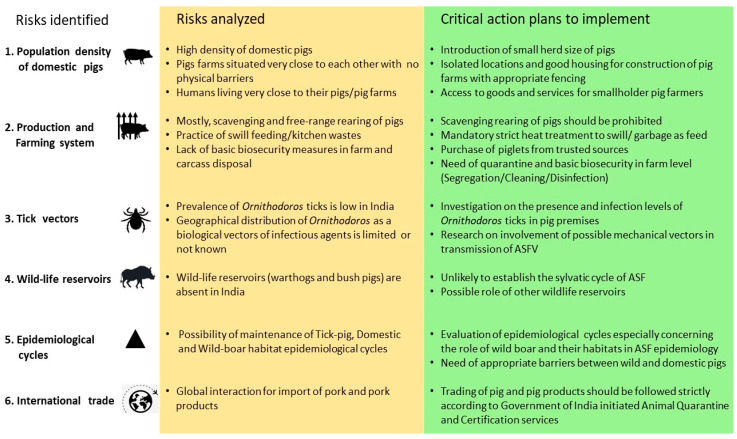
Risk identification and assessment of African swine fever and critical action plans to prevent future outbreaks.

The listed risk factors or indicators and the critical risk control measures to reduce or eliminate threat of future emergence of ASF in India are described in Figure 3. However, reviewing and updating the identified risks is an important future research for the global control and eradication of ASF.

## 6. Research Gaps

The most significant knowledge gaps in the prevention and control of ASF are (i) correlation of wild boar and ASF occurrence; (ii) ASFV survival and transmission; (iii) biosecurity; and (iv) surveillance [78]. To control ASF in countries where the wild boar habitat cycle persists, estimation of wild boar population in an area, correlation between wild boar and ASF occurrence, mechanism of ASFV spread and persistence in the wild boar population, and host to host transmission are required to be critically analyzed. With relation to ASFV survival and transmission, the role of vectors (soft ticks/biting flies) needs to be further investigated (particularly in the Indian scenario). Biosecurity measure is another significant gap in developing countries where pig production is a major source of economy. Livestock owners and farmers in villages and remote areas are not aware of biosecurity measures and being unable to identify the most effective measure for preventing infectious animal diseases bears severe economic loss. In such situations, veterinarians and para-veterinarians need to play a leading role to strengthen the veterinary services by educating the livestock owners about the disease and its prevention as well as carrying on consistent awareness programs on infectious disease management. Surveillance is another research gap with respect to ASF prevention and control and is considered as a high priority. To avoid or to limit future outbreaks, a strategic plan for control of ASF will be required for which we presume integrated (active and passive) surveillance is required as soon as possible. The major research gaps in ASF prevention are appropriately described by a study carried out by Alvarez and co-workers [78]. Apart from the mentioned research gaps identified for ASF, unavailability of a vaccine to date makes the disease difficult to control and once the disease is introduced into a pig population, culling of animals remain the only effective disease control option. 

## 7. Prevention and Control Strategies

African swine fever is devastating to the swine industry and currently, there is no commercially available vaccine to control the disease [79]. The development of vaccines against ASFV has been almost entirely neglected, mainly due to the technical difficulties involved in its development, gaps in knowledge concerning ASFV virulence factors [80], and to the fact that ASF was considered an ‘exotic’ disease in developed countries. However, the situation has dramatically changed with the recent emergence of the virus to Europe and Asia, threatening the global swine industry. Efforts to develop ASF vaccines are ongoing with different vaccine strategies. Among them, gene-deleted vaccines have shown promising results in eliciting effective immune responses [81,82,83,84,85,86]. In absence of effective vaccines, the need of implementing alternate measures of prevention is important and critical to control the disease.

### 7.1. Establish Control Zones

Zoning is one of the early actions to be taken when there is an incursion of ASF into a country. Zoning is the proclamation of geographical areas within a country into infected zones, surveillance zones, and free zones. To prevent further spread of infection, the infected zones should implement two objectives: (i) quarantine and livestock movement controls and (ii) remove sources of infection as quickly as possible through slaughter of potentially infected pigs, safe disposal of carcasses, and decontamination. Accordingly, active disease surveillance and preventing the entry of the disease by banning entry of pigs and pig products from the infected zones are actions which are associated with ASF surveillance and disease-free zones, respectively [87].

### 7.2. Establish Quarantine Facilities

Quarantine procedures to contain the disease, including pig-movement controls and prohibitions on the sale of potentially infected pig products, should be followed appropriately according to Government of India initiated Animal Quarantine and Certification Services. The quarantined period depends on the incubation period of a disease and usually a quarantine period of 3 weeks is set for observation of pig diseases [39].

### 7.3. Prohibition of Scavenging Pig Production Systems

The implementation of biosecurity measures in scavenging pig production systems is usually constrained by the producers’ limited capacity to invest resources and time, and by the nature of scavenging pig production [88]. In scavenging systems, measures related to introduction of new piglets from unknown sources, monitoring the health status, and unusual deaths of scavenger pigs, particularly regarding diseases of concern, should be given prime attention. Sometimes, a farmer may prefer to sell disease suspected animals to slaughter houses rather than undertaking control measures. The marketing of sick animals under scavenging rearing is a serious disease risk as these incubating or excreting sick animals disseminate disease very quickly to the healthy herd. Besides, practice of feeding untreated pig swill must be strictly avoided, which has been practiced for centuries in most of the developing and poor countries. Any unusual death of pigs should be immediately reported to veterinarians to know the biosecurity measures, proper disposal of animals, and disinfection of farm and premises. Even if disinfection is unlikely to be practicable, cleaning of night shelters/enclosures and equipment must be emphasized [88]. However, considering the biosecurity and socio-economic importance of pig farming in India, the practice of scavenging rearing should be immediately prohibited to prevent contact with other domestic pigs, wildlife, rodents, birds, and other livestock which creates favorable conditions for infectious disease spread.

### 7.4. Enchanced Biosecurity at Backyard and Small-Scale Farms

Biosecurity of pigs at backyard and small-scale farms should be effectively maintained to avoid the exposure of new or previously unknown pathogens in a farm, reduce the effect of endemic diseases in an area, and limit the circulation of infectious diseases to nearby farms. Segregation, cleaning, and disinfection are the major elements of biosecurity at the farm level. Implementation of quarantine measures of newly introduced animals, controlling the entry of pigs from outside farms/markets/villages, proper fencing of the farm area to prevent people, animals, and birds which may serve as mechanical vectors of ASF transmission, and maintaining adequate distances between farms are the important components of segregation [88]. Households with small scale pig farms are encouraged to routine clean pens and premises which includes quick removal of food, feces, litter, and dust, followed by disinfection using approved disinfectants.

### 7.5. Control Interstate Movement

Considering the first outbreak of ASF in Assam and Arunachal Pradesh states of India, prohibition of interstate movement of domestic pigs from the affected regions to a different part of the country should be given the highest priority as such movement may spread the infection to new geographical locations. Sub-acute and chronic forms of ASF might take 3–4 weeks of time to complete the incubation period. In such situations, pig vendors might take advantage of a long incubation period and could introduce infected stock quite successfully to buyers from a different state to disease-free zones. Therefore, development of systems to monitor the health status and to improve the traceability of relocated pigs (domestic and wild pigs) in order to enhance animal infectious disease surveillance, reporting, and response is advisable.

### 7.6. Ban/Control Illegal Import of Contaminated Pork and Pork Products

There is a greater chance of introduction of disease like ASF when contaminated pork and pork products are illegally imported from other countries for personal consumption or commercial purpose. Pathogens like ASFV are particularly challenging due to their prolonged infectiousness in pork products [89]. The porous international borders, particularly in the NE region of India, and migration of live pigs or illegal imports from the neighboring countries like Myanmar, Nepal, Bhutan, or China will make the country vulnerable for transboundary diseases [39]. Therefore, strict monitoring protocols should be sufficiently timed to detect transboundary movement of live animals or products to minimize the associated risk of hazards/pathogens.

### 7.7. Improve ASF Surveillance

Early disease detection is the key to maintain a sound animal health and is the most effective way to control ASF [90]. Laboratory diagnosis for detection of ASF as well differentiation of ASF with diseases with similar symptoms such as CSF, acute salmonellosis, swine erysipelas, porcine dermatitis and nephropathy syndrome, and Aujeszky’s disease should be implemented with proven and rapid diagnostic techniques to establish an accurate diagnosis within a short time. Polymerase chain reaction (PCR) is currently the most commonly used technique for the etiological diagnosis of ASF by amplifying the fragment of viral DNA present in the sample [7]. However, in developing countries like India, laboratories to detect such infectious diseases are often sparely distributed and access may be limited by economic or geographical factors. In such situations, the use of user-friendly kits or pen-side diagnostics (lateral flow assay, biosensors, mobile PCR assay, and isothermal assay techniques) [91] can have great potential to detect the disease in laboratory protocols. For the rapid detection of an antigen in the field, the use of economic and simple tools such as immunochromatographic strips are suitable alternate diagnostic devices essential for surveillance and control strategies [92]. It is also important to note that, veterinarians and livestock producers should be informed of the risk of ASF and the importance of reporting an outbreak by means of training and awareness camps by disease experts and professionals to ensure good field surveillance. Early disease detection, will therefore depend on the right balance between field surveillance and laboratory measures of disease detection.

### 7.8. Quick Elimination of Infected Animals and Proper Disposal

Pigs infected with ASF must be culled/slaughtered immediately using humane methods which should result in immediate death or immediate loss of consciousness lasting until death. For biosecurity considerations, infected animals should be killed first, followed by in-contact animals, and then the remaining animals. Different methods of humane killing have been adopted based on different age groups of animals. Neonatal pigs can be killed with free bullet, non-penetrative captive bolt, electrical applications, CO_2_/air mixture, nitrogen or inert gas mixed with CO_2_, and injection with barbiturates. Whereas, the adult pigs can be killed using free bullets, electrical applications, and injection with barbiturates [93]. The operational procedures of killing should be adopted, keeping in view the biosecurity and environmental aspects.

Disposal of carcasses should be carried out in such a way that the carcasses should no longer constitute a risk for further spread of the pathogen to the susceptible animals by direct or indirect means. In case of ASF, the method of safe disposal includes (i) rendering, (ii) incineration, (iii) burning, or (iv) deep burial on the spot [94]. Incineration or rendering is the most effective and easy way to dispose of carcasses. However, the movement of infected carcasses to the rendering plant may again pose a certain risk of spreading the disease and might not be feasible in Indian situations. Burning of carcasses in an outdoor area can also be done in several ways: pyre burning, pit burning, above-ground incineration, or a combination of the above methods. However, deep burial is a better option which can be done through trench burial or mass burial and in both the cases, the carcasses should be disinfected. Burial pits should be deep enough to ensure a soil layer of at least 1 m above the carcass to prevent scavenging and to avoid contamination [29].

### 7.9. Disinfection of Infected Premises

AFSV is extremely stable in the environment and is efficiently transmitted via contaminated blood and meat of infected animals. It can persist in excretions for approximately 3 days at 37 °C (3.71 and 2.88 days in feces and urine, respectively) [94], 11–22 days in viremic blood at 37 °C [95], over 84 to 155 days in raw meat stored under 4–8 °C [65], several months in boned meat and years in frozen carcasses at 4–8 °C [96].

Therefore, decontamination of animal houses, sheds, pens, yards, water-troughs, and nearby areas is extremely important to reduce the risk of contaminating the environment with the ASFV. Appropriate disinfectants that are effective in inactivation of ASFV include 2% sodium hydroxide, detergents and phenol substitutes, sodium or calcium hypochlorite (2–3% chlorine), and iodine compounds [87,97].

### 7.10. Investigations on Prevalence of Soft Ticks

Distribution of argasid or soft ticks can be found throughout the world, except for places with extreme climatic conditions [50,98]. However, the distribution of specific species of soft tick is more limited or may be very extensive, depending on factors such as the adaptability of each particular species to new ecological environments, the dissemination of immature phases by migratory birds, and the ability of adult specimens to infest different host species [50]. Considering the involvement of Ornithodoros ticks as a biological vector of ASF, it is also possible that species that have never been identified in India may be imported from other continents, contributing to a geographic distribution of particular species in constant evolution. Therefore, an extensive investigation on the prevalence of Ornithodoros species is important to know the status of their distribution throughout the country and should be included in control strategies.

Along with the critical preventive and control measures mentioned for ASF, public awareness and education campaigns are important integral elements of the disease control strategies. Disease awareness campaigns should be targeted in pig rearing areas in India because rural farmers lack knowledge on animal diseases, clinical symptoms, and biosecurity measures. Exotic diseases like ASF should be given equal importance as human diseases when it comes to the socio-economic status of developing nations like India since a majority of the population depends on agriculture and livestock farming for food security and livelihood. 

## 8. Conclusions

ASF in India has probably emerged through a neighboring country, that shares its border with India. Genetic characterization and molecular evolutionary analysis to trace the ancestors of the present outbreak are ongoing. As per the recent statement from OIE, outbreaks of ASF in India lead to a current count of over 3700 dead pigs in affected regions [9]. The current statistics and data on the number of pigs that died due to the disease are still undergoing revisions and will be updated soon by Animal Husbandry departments of Arunachal Pradesh and Assam states of India. In the present study, we identified the underlying risk factors that might favor future outbreaks of ASF in the Indian pig population. On qualitative risk assessment analysis, we observed that the identified risk factors are well preserved in Indian geography and might participate in future outbreaks in the country or disseminating the disease to nearby countries. Besides, lack of a highly organized veterinary structure and effective traceability system for animals in India makes the control of ASF a big challenge. Therefore, the described risk factors should be critically considered and each risk factor should be reassessed at a regional level. Immediate containment measures should be implemented to control the disease, followed by establishing a strategic research plan on surveillance and sero-epidemiology of ASF on domestic and wild pig population. Studies on the involvement of biological vectors in the perpetuation and transmission of ASFV is also important to estimate these risks more accurately in the Indian scenario.
